# Targeting EZH2 reverses thyroid cell dedifferentiation and enhances iodide uptake in anaplastic thyroid cancer

**DOI:** 10.1002/1873-3468.70207

**Published:** 2025-10-28

**Authors:** Diego Claro de Mello, Marcella Maringolo Cristovão, Guilherme Henrique, Vinicius Gonçalves Rodrigues, Caroline Serrano‐Nascimento, Edna Teruko Kimura, Cesar Seigi Fuziwara

**Affiliations:** ^1^ Department of Cell and Developmental Biology, Institute of Biomedical Sciences University of São Paulo (USP) Brazil; ^2^ Department of Medicine, Laboratory of Molecular and Translational Endocrinology Medicine Federal University of São Paulo (UNIFESP) Brazil; ^3^ Department of Biological Sciences, Institute of Environmental, Chemical and Pharmaceutical Sciences (ICAQF) Federal University of São Paulo (UNIFESP) Diadema Brazil

**Keywords:** anaplastic thyroid cancer, EZH2, iodide uptake, NIS, Tazemetostat, thyroid differentiation

## Abstract

Anaplastic thyroid carcinoma (ATC) is a highly aggressive malignancy characterized by dedifferentiation and radioiodine refractoriness. We investigated whether EZH2‐mediated H3K27me3 deposition represses thyroid differentiation genes (TDGs) in ATC cells. Online ChIP‐seq analyses and CUT&RUN confirmed EZH2/H3K27me3 enrichment at key TDGs (*SLC5A5*, *NKX2‐1*, *TSHR*, *FOXE1*, *TPO*). Pharmacological inhibition of EZH2 with EPZ6438 reactivated TDG expression in RAS and BRAF‐mutated ATC cell lines and partially restored iodide uptake. Co‐treatment with the MEK1/2 inhibitor U0126 further enhanced TDG expression, consistent with MAPK‐dependent regulation of EZH2. These findings reveal EZH2 as a mediator of ATC dedifferentiation and highlight its inhibition as a potential strategy to restore thyroid function and sensitize tumors to radioiodine.

**Impact statement**

This study reveals how EZH2‐driven epigenetic remodeling controls thyroid cell dedifferentiation and loss of iodide uptake in anaplastic thyroid cancer. Our findings provide new mechanistic insights and highlight an FDA‐approved drug with repurposing potential, advancing both anaplastic thyroid cancer biology research and therapeutic perspectives.

## Graphical abstract

Anaplastic thyroid cancer (ATC) lacks iodide uptake ability due to MAPK activation increasing the expression of the histone methyltransferase EZH2, which represses thyroid differentiation genes (TDGs) such as the sodium iodide symporter (NIS). Dual inhibition of MAPK (U0126) and EZH2 (EPZ6438/Tazemetostat) reverses this mechanism, thus restoring TDG expression, including the expression of NIS and thyroid‐stimulating hormone receptor, to significantly enhance iodide uptake.

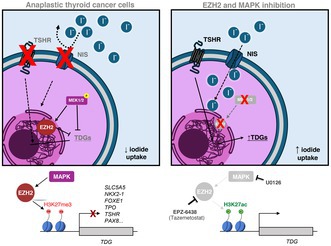

## Abbreviations

**a.u.**, arbitrary units

**ATC**, anaplastic thyroid carcinoma

**bp**, base pairs

**BRAF**, B‐Raf proto‐oncogene, serine/threonine kinase (V600E)

**ChIP‐qPCR**, chromatin immunoprecipitation followed by quantitative PCR

**ChIP‐seq**, chromatin immunoprecipitation sequencing

**CTR**, control

**CUT and RUN**, cleavage under targets and release using nuclease

**DIO1**, iodothyronine deiodinase 1

**DIO2**, iodothyronine deiodinase 2

**DMSO**, dimethyl sulfoxide

**DUOX1**, dual oxidase 1

**DUOX2**, dual oxidase 2

**E + U**, EPZ6438 + U0126 (Combined inhibition)

**EED**, embryonic ectoderm development

**EPZ**, EPZ6438/Tazemetostat (EZH2 activity inhibitor)

**ERK1/2**, extracellular signal‐regulated kinase 1/2

**EZH2**, enhancer of zeste homolog 2

**FBS**, fetal bovine serum

**FDA**, US Food and Drug Administration

**FOXE1**, forkhead box E1

**FTC**, follicular thyroid carcinoma

**GLIS3**, GLIS family zinc finger 3

**H3K27ac**, acetylation of lysine 27 on histone H3

**H3K27me3**, trimethylation of lysine 27 on histone H3

**HDAC**, histone deacetylase

**HHEX**, hematopoietically expressed homeobox

**I** ^**−**^, iodide

**IgG**, immunoglobulin G

**iPSC**, induced pluripotent stem cell

**kDa**, kilodalton

**MAPK**, mitogen‐activated protein kinase

**MEK1/2**, mitogen‐activated protein kinase kinase 1/2

**NIS**, sodium iodide symporter

**NKX2‐1**, NK2 homeobox 1

**PAX8**, paired box 8

**PD‐L1**, programmed death‐ligand 1

**pERK**, phosphorylated ERK (extracellular signal‐regulated kinase)

**pk**, peak

**PRC2**, polycomb repressive complex 2

**PTC**, papillary thyroid carcinoma

**qPCR**, quantitative PCR

**RAS**, RAS GTPase proto‐oncogenes (HRAS and NRAS)

**RP score**, regulatory potential score

**RPL19**, ribosomal protein L19

**SAHA**, suberoylanilide hydroxamic acid

**SD**, standard deviation

**SLC16A2**, solute carrier family 16 member 2 (MCT8)

**SLC26A4**, solute carrier family 26 member 4 (pendrin)

**SLC26A7**, solute carrier family 26 member 7

**SLC5A5**, solute carrier family 5 member 5

**SUZ12**, SUZ12 polycomb repressive complex 2 subunit

**TDGs**, thyroid differentiation genes

**TG**, thyroglobulin

**TPO**, thyroid peroxidase

**TSH**, thyroid‐stimulating hormone

**TSHR**, thyroid‐stimulating hormone receptor

**TSS**, transcription start site

**U0**, U0126 (MEK1/2 inhibitor)

**VCP**, valosin‐containing protein

**WDTC**, well‐differentiated thyroid cancers

Anaplastic thyroid cancer (ATC) is the rarest variant (1–3%) but the major cause of thyroid cancer‐related deaths [1, 2]. ATC can arise *de novo* or may progress from well‐differentiated thyroid cancers (WDTC), which have an associated overall patient survival rate of ~ 90% [3]. In contrast to WDTC, ATC patients' prognosis is still poor (less than 1 year) as ATC is highly metastatic, unresectable, and resistant to radioiodine therapy due to thyroid cell dedifferentiation [1, 4]. Thyroid dedifferentiation is marked by the transcriptional silencing of genes essential for thyroid hormone synthesis and iodide uptake, leading to loss of key functional markers, such as the sodium iodide symporter (NIS) [5].

Uptake of iodide in thyroid follicular cells is mediated by NIS (encoded by *SLC5A5*), a glycoprotein located at the basolateral membrane of thyrocytes, which cotransports two sodium cations and one iodide anion into the cell. Once internalized, iodide is oxidized to iodine by thyroid peroxidase (TPO) and incorporated into thyroglobulin (TG) to produce thyroid hormones. Thyroid‐stimulating hormone (TSH) is a key regulator of NIS expression and driver of thyroid hormone synthesis through its receptor (TSHR) [6]. In WDTC, thyroidectomy combined with TSH stimulation and radioactive iodide therapy is generally effective [7, 8]. However, in poorly differentiated carcinomas and ATC, radioiodine refractoriness is frequent due to transcriptional silencing of thyroid differentiation genes (TDGs) and impaired SLC5A5/NIS trafficking to the plasma membrane, both hallmarks of thyroid dedifferentiation [8]. These characteristics are often associated with complex mutational and epigenetic landscapes in ATC.

Among the molecular alterations, *TERT* promoter and *TP53* mutations are the most frequent in ATC (> 70%), along with driver mutations inherited from WDTC precursors, most commonly BRAF^V600E^ and RAS mutations (NRAS^Q61R^ and HRAS^G13R^) [9]. Importantly, the clinical impact of these mutations varies, as patients with BRAF^V600E^‐mutated ATC have benefited from the recently approved combination of dabrafenib (BRAF inhibitor) and trametinib (MEK1/2 inhibitor) [10], whereas patients with RAS‐mutated ATC currently lack effective targeted therapies and exhibit poorer outcomes [11, 12, 13]. However, besides the mutational burden, the profound dedifferentiation in tumor cells and consequent therapy refractoriness observed in ATC point to additional mechanisms such as epigenetic modifications as contributors to TDGs repression and tumor aggressiveness.

One such mechanism involves overactivation of EZH2, the catalytic subunit of the Polycomb Repressive Complex 2 (PRC2), which mediates trimethylation of lysine 27 on histone H3 (H3K27me3), a posttranslational histone modification associated with transcriptional repression [14]. Previous work from our group indicates that PRC2 components (EZH2, EED, and SUZ12) are overexpressed in ATC, and targeting of EZH2 in BRAF‐mutated ATC cell lines with the FDA‐approved drug EPZ6438 (Tazemetostat) or with CRISPR/Cas9 improved gene expression of TDGs and inhibited cell migration, invasion, and *in vivo* tumor growth [15]. In the context of EZH2‐mediated dedifferentiation, EZH2 represses PAX8 gene expression by targeting its promoter in ATC [16], and in papillary thyroid carcinoma (PTC) cells, a variant of WDTC, Tazemetostat treatment enhances TDGs expression and iodide uptake [17]. These observations suggest that modulating EZH2 may help restore thyroid differentiation and counteract ATC aggressiveness. However, the precise mechanisms by which EZH2 drives dedifferentiation and aggressive behavior, especially across different mutational contexts, remain unclear.

Here, we report that targeting EZH2 methyltransferase activity with Tazemetostat significantly improves TDGs expression and iodide uptake in both BRAF‐mutated and RAS‐mutated ATC cell lines. Moreover, we demonstrate that EZH2 promotes transcriptional silencing of key thyroid‐related genes, including *SLC5A5*, *TPO*, *TSHR*, *FOXE1*, and *NKX2‐1*, through H3K27me3 deposition. These findings suggest EZH2/PRC2 as a major epigenetic regulator mediating ATC dedifferentiation, and its inhibition may offer a promising therapeutic strategy for enhancing iodide uptake and reversing thyroid cell dedifferentiation.

## Materials and methods

### Cell culture and treatments

We used a panel of ATC cell lines, Hth7, C643, KTC2, and SW1736, in all experiments. The additional cell lines and corresponding cell culture medium, histology, genetic drivers, and identifiers are shown in Table 1. All cells were kept in a humidified incubator at 37 °C with 5% CO_2_. SW1736, C643, Hth7, PCCl3, TPC1, and BCPAP cell lines were kindly provided by Dr James Fagin (Memorial Sloan Kettering Cancer Center, USA). KTC2 cell line was kindly provided by Dr Norisato Mitsutake (University of Nagasaki, Japan). Nthy‐ori 3.1 and K1 cell lines were obtained from the European Collection of Authenticated Cell Cultures (ECACC) repository *via* Sigma‐Aldrich.

**Table 1:** Cell lines used and culture conditions. FBS, Fetal bovine serum; RRIDs, resource identification initiative; TSH, thyroid‐stimulating hormone.

Cell line	Histology	Genetic driver	Culture medium	RRIDs
C643	ATC	HRAS^G13R^	RPMI1640 + 10% FBS	CVCL_5969
Hth7	ATC	NRAS^Q61R^	DMEM + 10% FBS + 1% Glutamine (100×)	CVCL_6289
KTC2	ATC	BRAF^V600E^	RPMI1640 + 5% FBS	CVCL_6476
SW1736	ATC	BRAF^V600E^	RPMI1640 + 10% FBS	CVCL_3883
Nthy‐ori 3.1	Nontumoral human thyroid follicular cells	*SV40*	RPMI + 10% FBS + 1% Glutamine (100×)	CVCL_2659
PCCl3	Normal rat thyroid follicular cells	–	F‐12 Coon's + 5% FBS + 1 mU·mL^−1^ bovine TSH, 10 μg·mL^−1^ insulin, 5 μg·mL^−1^ transferrin, and 10 nm hydrocortisone	CVCL_6712
TPC‐1	PTC	RET/PTC1	DMEM +5% FBS	CVCL_6298
K1	PTC	BRAF^V600E^	DMEM + 10% FBS + 1% Glutamine (100×)	CVCL_2537
BCPAP	PTC	BRAF^V600E^	DMEM + 10% FBS	CVCL_0153

We confirm that all the cell lines used in this study were authenticated by short tandem repeat (STR) analysis following the ANSI Standard ASN‐0002 (2012), and the results were compared with the DSMZ and Cellosaurus databases. Briefly, 22 STR loci plus the gender‐determining locus Amelogenin were amplified using the commercially available PowerPlex® Fusion 6C Kit (Promega, Madison, WI, USA). Samples were processed on an ABI 3130 Genetic Analyzer (Thermo Fisher Scientific, Waltham, MA, USA), and data were analyzed with GeneMarker® hid software (SoftGenetics, State College, PA, USA). In addition, all experiments were performed using mycoplasma‐free cells.

For gene expression, western blots, and iodide uptake assays, cells were treated with 5 μm EPZ6438 (Tazemetostat‐Cayman Chemical, Ann Arbor, MI, USA), an allosteric inhibitor of EZH2, alone or combined with 10 μm U0126 (Promega), a MEK1/2 inhibitor. The EZH2 inhibitor was added to the medium every 2 days (6 days treatment), and on days 4 and 5, the MEK1/2 inhibitor was added (48 h total treatment). DMSO was used as a drug diluent and as a vehicle control.

This study did not involve direct experimentation on human or animal subjects. Instead, it utilized commercially available human and rat cell lines and publicly accessible repositories for ChIP‐seq data from human cell lines. Therefore, no ethical approval was required.

### Cistrome data browser

We accessed the Cistrome data browser [18] (http://cistrome.org/db/, version 2.0; 12/12/2024) to obtain the regulatory potential score (RP score) using EZH2 and H3K27me3 as factors to analyze genomic regions of thyroid differentiation genes and thyroid‐specific transcription factors for ChIP‐seq data in non‐thyroid cells using the criteria as previously described [15]. For EZH2 enrichment data, the following Cistrome ID studies were utilized: 74684 (Erythroblast), 41794 (iPSC), 41788 (iPSC), 90091 (ESC), and 8664 (Prostate Cancer). For H3K27me3 enrichment data, the following Cistrome ID studies were utilized: 45141 (Lung Cancer), 71329 (Fibroblast), 71328 (Fibroblast), 52394 (Melanoma), and 52389 (Melanoma). To compare peaks for EZH2/H3K27me3 and for primer design for CUT&RUN and ChIP‐qPCR assays, we used as a control the acetylation marks (H3K27ac) obtained from a normal thyroid tissue (Cistrome study ID 102556). A positive regulation was considered if the RP score > 1.

### Chromatin immunoprecipitation

SW1736 cells (4 × 10^6^ per ChIP) were used for ChIP with EZH2 and H3K27me3 antibodies (Cell Signaling, Danvers, MA, USA). The ChIP assay was performed according to the Cell Signaling SimpleChIP® Plus Chromatin Immunoprecipitation Protocol (Magnetic Beads). Chromatin was cross‐linked with methanol‐free formaldehyde (Thermo Fisher) to preserve DNA‐protein interactions. Next, the nuclei were pelleted, and chromatin was digested with micrococcal nuclease at 37 °C for 20 min. After that, nuclei were disrupted with sonication using a Sonics Vibracell 130‐Watt ultrasonic processor (VCX 130PB) (Newton, CT, USA) with 3 pulses of 20 s at 40% amplitude to release the cross‐linked, digested chromatin. A 2% input was separated at this stage to be used as qPCR control (10 μL from a 500 μL nuclei lysate). Next, the immunoprecipitations were performed by incubating chromatin samples (10 μg per ChIP) with 5 μL of anti‐EZH2, anti‐H3K27me3, and anti‐Histone H3 for the positive control or anti‐Rabbit (Cell Signaling) for the negative control overnight at 4 °C under rotation in a HulaMixer™ (Thermo Fisher, Scotland, UK). After incubation, protein G magnetic beads were added to separate antibodies that interacted with target protein/DNA from the total cross‐linked lysate, with incubation at 4 °C for 2 h. After washing in salted solutions, chromatin was eluted from the magnetic beads and decross‐linked in 1× ChIP Elution Buffer containing 200 μm NaCl and proteinase K, with overnight incubation at 65 °C. Next, DNA was purified with spin columns and used in qPCRs with SYBR Green Master Mix (Thermo Fisher) to detect enrichment in *SLC5A5* and *NKX2‐1* regulatory regions using specific primers (Table 2). Percent input was calculated by comparing IP samples to the 2% input. The qPCR products were processed on 2% agarose gels and visualized on a UV transilluminator ImageQuant LAS4000 imaging system (GE Healthcare, Little Chalfont, UK).

**Table 2:** List of primers used for ChIP‐qPCR.

Target^a^	Sequence 5′–3′	Amplicon size	Concentration
SLC5A5_PK3 FW (−13)	ACAATCACGAGCTGCTCCCG	171 bp	200 nm
SLC5A5_PK3 RV (+158)	CGCTGTCTGTCTCTGCGTCC		
SLC5A5_PK4 FW (+717)	TCTTCTACCGCCTGGGCCTC	142 bp	200 nm
SLC5A5_PK4 RV (+865)	CCATCCGCGTCCTCCTGTAC		
NKX2‐1_PK3 FW (+15)	TGACAGACACGTAGACCAAC	220 bp	200 nm
NKX2‐1_PK3 RV (+234)	GACAGGTCTTTAGGAGGAGG		
NKX2‐1_PK4 FW (+2469)	TTCCAGAACCACCGCTACAA	169 bp	200 nm
NKX2‐1_PK4 RV (+2638)	TCACCAGGACCGGCACCGCC		

^a^ Flanked region according to distance from the transcription start site (TSS) is shown in parentheses.

### CUT&RUN

Cleavage under Targets and Release Using Nuclease (CUT&RUN) assay was performed using the CUT&RUN Assay Kit (#86652) from Cell Signaling Technologies. One hundred thousand SW1736 cells per immunoprecipitation (IP), including positive and negative antibody controls and a nonprecipitated control (input), were harvested by trypsinization. After washing the cells, each IP was incubated with 10 μL of Concanavalin A beads for 5 min at room temperature. After incubation, the beads were resuspended in antibody binding buffer and incubated overnight at 4 °C with the primary antibodies: 2 μL of anti‐H3K27me3 (C3B11), anti‐EZH2 (D2C9), anti‐H3K4me3 (9751F – positive control), and 5 μL of anti‐IgG (663625 – negative control). Following antibody incubation, the IPs were washed with permeabilization buffer and incubated with pAG‐MNase enzyme for 1 h at 4 °C. The pAG‐MNase enzyme was then activated with calcium chloride for 30 min at 4 °C, and the reaction was stopped using a buffer containing RNase and 5 μL of spike‐in (36S98S) for DNA normalization. The final supernatant (chromatin‐enriched) was transferred into new 2 mL tubes. For the input sample, chromatin digestion was performed using the Micrococcal Nuclease protocol (#10011, Cell Signaling), as previously described, followed by sonication with three 20‐s pulses at 40% amplitude for DNA fragmentation. The DNA was then purified using spin columns (Qiagen, Hilden, Germany) and analyzed in qPCRs with SYBR Green Master Mix (Thermo Fisher) to detect enrichment in the *SLC5A5*, *NKX2‐1*, *TSHR*, *FOXE1*, and *TPO* regulatory regions using specific primers (Table 3). Additionally, spike‐in (#85756P) and RPL30 (#7014P) primer sets were included for DNA normalization and to serve as a positive control for the assay, respectively. Enrichment was calculated using the following formula: $\left(\text{Percent Input}=100\%\times 2^{\left(C\left[T\right]\;100\%\;\text{Input Sample}-C\left[T\right]\;\mathrm{IP}\;\text{Sample}\right)}\right)$. Enrichment values were then normalized using spike‐in control to calculate the normalization factor. The qPCR products were processed on 2% agarose gels and visualized on a UV transilluminator ImageQuant LAS4000 imaging system (GE Healthcare, Little Chalfont, UK).

**Table 3:** List of primers used for CUT&RUN‐qPCR.

Target^a^	Sequence 5′–3′	Amplicon size	Concentration
SLC5A5_PK1 FW (+505)	CTGAGGACTTCTTCACCGGG	77 bp	500 nm
SLC5A5_PK1 RV (+582)	CGACATGAAGCTGGCAGACA		
SLC5A5_PK2 FW (+683)	GCTCTTCATGCCCGTCTTCT	63 bp	500 nm
SLC5A5_PK2 RV (+746)	CCTCTGTCCGGTACCTCGTA		
NKX2‐1_PK1 FW (+16)	ACGTAGACCAACAGTGCGG	66 bp	500 nm
NKX2‐1_PK1 RV (+82)	GCTCATTTGTTGGCGACTGG		
NKX2‐1_PK2 FW (+2422)	CACCGGGTGCCCGCAGCAGC	71 bp	500 nm
NKX2‐1_PK2 RV (+2493)	GCGCTTCATTTTGTAGCGGT		
TSHR_PK1 FW (−420)	GATAAGGAGTGCGTGCGAGT	69 bp	500 nm
TSHR_PK1 RV (−351)	CACCTTCCCTAGCGTGTAGC		
TSHR_PK2 FW (+191)	TCAACGCATCCCCAGCTTAC	60 bp	500 nm
TSHR_PK2 RV (+251)	TGATCTCTCCCGGGTACTCA		
FOXE1_PK1 FW (+275)	AATCCTAAACTAGCGGGCACC	71 bp	500 nm
FOXE1_PK1 RV (+346)	CGAGGGGTCCGGAAGTGA		
FOXE1_PK2 FW (+1000)	CCTCACACTCAACGACTGCT	70 bp	500 nm
FOXE1_PK2 RV (+1070)	CCAGTAGTTGCCCTTACCCG		
TPO_PK2 FW (+99 273)	CCCAGTGCTACCTCTTTGCT	61 bp	500 nm
TPO_PK2 RV (+99 336)	TGGAGAGGCAGATGGACAGT		

^a^ Flanked region according to distance from the transcription start site (TSS) is shown in parentheses.

### Western blot

Total protein was extracted from cells using RIPA buffer (20 mm Tris, pH 7.5, 150 mm NaCl, 1% Nonidet P‐40, 0.5% sodium deoxycholate, 1 mm EDTA and 0.1% SDS) containing 10% protease inhibitor cocktail and 1% phosphatase inhibitor cocktail (Sigma, St. Louis, MO, USA). Pellets were sonicated using a 3 V pulse for 10 s and protein concentration was determined using the Bradford assay (Bio‐Rad Laboratories, Hercules, CA, USA). For western blots, 20 μg of each sample was fractionated by 10% SDS/PAGE and blotted onto a nitrocellulose Hybond‐ECL membrane (Amersham Biosciences, Little Chalfont, UK). Nonspecific binding sites were blocked with 5% skim milk in Tris‐buffered saline – 0.1% Tween‐20. The primary antibodies used are shown in Table 4, and incubations were performed overnight at 4 °C with gentle mixing. Antibody bound to target protein was detected with horseradish‐peroxidase‐conjugated secondary antibodies and developed using luminol and p‐coumaric acid (Sigma) reagents in the presence of hydrogen peroxide. Chemiluminescence emission was visualized with an imagequant las4000 imaging system (GE Healthcare, Little Chalfont, UK). For quantification, we used the imagej software.

**Table 4:** Primary antibodies used for western blots. BSA, bovine serum albumin.

Antibody	Source	Dilution^a^
Mouse anti‐β‐Actin (sc‐4778)	Santa Cruz (Dallas, TX, USA)	1 : 1000/skim milk
Rabbit anti‐ERK1/2 (sc‐94)	Santa Cruz	1 : 1000/skim milk
Rabbit anti‐phospho Erk1/2 (D13.14E)	Cell Signaling	1 : 2000/BSA
Rabbit anti‐H3K27me3 (C3B11)	Cell Signaling	1 : 1000/BSA
Rabbit anti‐H3 (D2B12)	Cell Signaling	1 : 2000/skim milk
Rabbit anti‐EZH2 (D2C9)	Cell Signaling	1 : 1000/skim milk

^a^ All dilutions were performed in Tris‐buffered saline (TBS) containing 0.1% Tween‐20 and 5% skim milk or BSA.

### Gene expression analysis

Total RNA was extracted using the phenol–chloroform method with the TRIzol reagent (Invitrogen‐Thermo Fisher, Carlsbad, CA, USA). The reverse transcription of 1 μg of total RNA was performed using oligo‐dT primer and MMLV reverse transcriptase (Invitrogen, Thermo Fisher Scientific, Waltham, MA, USA). The expression of the thyroid differentiation genes was analyzed by quantitative PCR (qPCR) using SYBR Green Master Mix, cDNA, and specific primers (Table 5) in a ViiA7® Sequence Detection System (Applied Biosystems, Thermo Fisher Scientific, Waltham, MA, USA). Gene expression was normalized by comparison with *RPL19* levels and calculated using the QGene program [19] using Ct data.

**Table 5:** List of primers used for qPCR.

Target	Sequence 5′–3′	Amplicon size	Concentration
SLC5A5 FW	AGTACATTGTAAGCCACGATGCTGTA	76 bp	200 nm
SLC5A5 RV	CGGTCACTTGGTTCAGGATGA		
TG FW	CCTGCTGGCTCCACCTTGTTT	352 bp	200 nm
TG RV	CCTTGTTCTGAGCCTCCCATCGTT		
TPO FW	ACGCCTCTGCGAGGTGC	189 bp	200 nm
TPO RV	TGCAAATCACCGTCGAGGT		
TSHR FW	CCTTCACCTCACACGGGCT	107 bp	200 nm
TSHR RV	TGCTCTCATTACACATCAAGGACTC		
NKX2‐1 FW	CAGCCTGTCCCACCTGAACT	65 bp	200 nm
NKX2‐1 RV	ATAGCAAGGTGGAGCAGGACAT		
PAX8 FW	GGCATGGTGGCAGGAAGT	78 bp	200 nm
PAX8 RV	GCGCCAGGCCTCGCTGTAGGA		
HHEX FW	TGCATAAAAGGAAAGGCGGC	208 bp	200 nm
HHEX RV	TTGCTTTGAGGGTTCTCCTGT		
FOXE1 FW	TGAGCCAGCGTAGGGACGAAAA	191 bp	200 nm
FOXE1 RV	CCACCTCCTCCCGTTTACAGAGTA		
DUOX2 FW	GCTGCCTTCCCTTAGTGAGT	236 bp	400 nm
DUOX2 RV	CACACGGAGCACAGTGAGAT		
GLIS3 FW	AACGCCCGCTATAAACTGCT	81 bp	200 nm
GLIS3 RV	CTCGCAACCTTCAAACGTACA		
SLC26A4 FW	ACTGCTGGATTGCTCACCAT	201 bp	400 nm
SLC26A4 RV	TTCAGGAGGCAAAAACCCCC		
SLC26A7 FW	TGTACAGCACAGGAGCGAAG	119 bp	400 nm
SLC26A7 RV	GCTTGCAAGGACACACATGG		
RPL19 FW	TCTCATGGAACACATCCACAA	139 bp	200 nm
RPL19 RV	TGGTCAGCCAGGAGCTTCTT		

### *In vitro* nonradioactive iodide uptake

NIS‐mediated iodide uptake was evaluated using the Sandell‐Kolthoff (SK) reaction protocol [20]. Briefly, 5 × 10^4^ cells were seeded in 24‐well plates. The following day, cells were incubated with 25 μm NaI (Sigma‐Aldrich, St. Louis, MO, USA) for 45 min. After washing with PBS, 100 μL of dH_2_O was added per well and shaken for 10 min. Afterward, 80 μL was transferred to a 96‐well plate, and 200 μL of 0.2 N arsenious acid was added to each well. The plate was shaken for 15 min before the addition of 30 μL of ammonium cerium(IV) sulfate solution. The reaction mixture was shaken for 40 min at room temperature. Absorbance was measured at 415 nm, and iodide uptake was quantified using a standard curve.

### Statistical analysis

The results are presented as mean ± standard deviation (SD) and were submitted to analysis of variance followed by a *t*‐test or the Tukey test using graphpad prism (GraphPad Software, Boston, MA, USA). Differences were considered significant at *P* ≤ 0.05.

## Results

### EZH2 inhibition improves thyroid function by improving cell differentiation

We first assessed EZH2 protein levels across a panel of thyroid cancer cell lines. RAS‐mutated ATC cell lines (Hth7 and C643) and BRAF‐mutated ATC cell lines (KTC2 and SW1736) exhibited markedly higher EZH2 expression compared to PTC‐derived cell lines (BCPAP, TPC1, and K1) (Fig. S1).

To evaluate whether EZH2 inhibition alone or in combination with MEK1/2 inhibition could restore thyroid‐specific gene expression and function, we treated the RAS‐mutant Hth7 (NRAS^Q61R^) and C643 (HRAS^G13R^) cell lines with EPZ6438 (an allosteric inhibitor of EZH2) for 6 days at 5 μm, either alone or combined with 10 μm of U0126 (selective inhibitor of MEK1/2), added during the last 48 h of treatment.

**Fig. 1:**
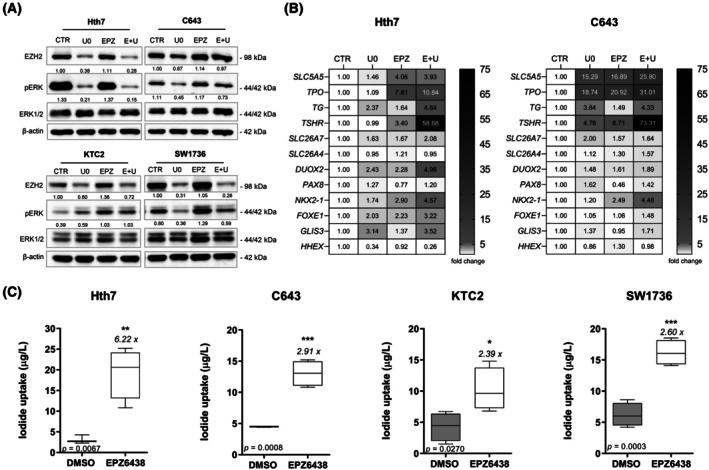
EZH2 inhibition enhances thyroid gene expression and iodide uptake in ATC cells. (A) Western blot of EZH2, phosphorylated ERK (pERK), total ERK1/2, and β‐Actin (loading control) in RAS‐mutated (Hth7, C643) and BRAF‐mutated (KTC2, SW1736) cells treated for 6 days with U0126 (U0, MEK1/2 inhibitor), EPZ6438 (EPZ, EZH2 activity inhibitor), or the combination (E + U). (B) Heatmap representation of qPCR results showing the relative mRNA expression of 11 thyroid differentiation genes (*SLC5A5*, *TPO*, *TG*, *TSHR*, *SLC26A7*, *SLC26A4*, *DUOX2*, *PAX8*, *NKX2‐1*, *FOXE1*, *GLIS3*, and *HHEX*) in Hth7 and C643 cells following the indicated treatments. Values are expressed as fold‐change vs. DMSO‐treated controls (CTR). Data are representative of two independent experiments performed in triplicate. (C) Non‐radioactive iodide uptake assay in RAS‐mutated (Hth7, C643) and BRAF‐mutated (KTC2, SW1736) cells after 6 days of EPZ6438 treatment. Box plots represent mean ± SD from two independent experiments. Fold‐change increase, statistical significance, and *P*‐values (from *t‐*tests) are indicated. *P < 0.05 vs DMSO; **P < 0.01 vs DMSO; ***P < 0.001 vs DMSO.

Interestingly, MEK1/2 inhibition led to a reduction in EZH2 expression in Hth7, KTC2, and SW1736 cells, suggesting that EZH2 levels are at least partially regulated by MAPK pathway activation (Fig. 1A). Conversely, targeting the methyltransferase of EZH2 with EPZ6438 led to an increase in phosphorylated ERK levels in KTC2 and SW1736 cells, an effect that was partially reversed upon combined inhibition of EZH2 and MEK1/2 (Fig. 1A).

Next, we investigated whether EZH2 and/or MAPK inhibition could enhance expression of TDGs in RAS‐mutated cell lines. Using qPCR, we analyzed the expression of TDGs. EZH2 inhibition alone significantly upregulated the expression of *SLC5A5*, *TPO*, *TSHR*, and *NKX2‐1* in both cell lines (Fig. 1B, Fig. S2). Notably, C643 cells (HRAS‐mutated) were more responsive to MEK1/2 inhibition, showing increased expression of additional TDGs, including *TG*, *PAX8*, and *GLIS3*. Moreover, the combination of EZH2 and MEK1/2 inhibition had a synergistic effect, further enhancing *TSHR*, *NKX2‐1*, and *FOXE1* expression in both cells. To compare these results, we also analyzed the BRAF‐mutated cell lines KTC2 and SW2736, which our group previously reported to exhibit increased TDG expression following EZH2 and MEK1/2 inhibition [15] (Fig. S3).

We then asked whether EZH2 inhibition could restore the defining functional property of thyroid cells in iodide uptake. Using a non‐radioactive iodide uptake assay, we observed that EPZ6438 treatment significantly increased iodide uptake in all tested cell lines. In RAS‐mutated Hth7 and C643 cells, iodide uptake increased by 6.22‐fold and 2.91‐fold, respectively; in BRAF‐mutated KTC2 and SW1736 cells, uptake increased by 2.39‐fold and 2.60‐fold, respectively (Fig. 1C). Although these results indicate partial functional restoration, iodide uptake levels remained below those of well‐differentiated models, reaching approximately 50% of the uptake observed in the PTC cell line BCPAP and ~ 15% compared to the normal human thyroid cell line Nthy‐ori 3.1 (Fig. S4).

### EZH2 represses thyroid differentiation genes and transcription factors *via* H3K27me3 deposition

Having explored the pivotal role of EZH2 in thyroid dedifferentiation, we investigated whether EZH2 and its associated repressive histone mark H3K27me3 directly contribute to the epigenetic silencing of thyroid‐specific genes in ATC.

We first analyzed publicly available ChIP‐seq datasets for EZH2 and H3K27me3 from non‐thyroid cell types using the Cistrome DB. To predict EZH2/PRC2 binding regions relevant to thyroid differentiation, we compared these datasets to H3K27ac peaks from normal thyroid tissue. These bioinformatic predictions were then experimentally validated by CUT&RUN and ChIP‐qPCR.

**Fig. 2:**
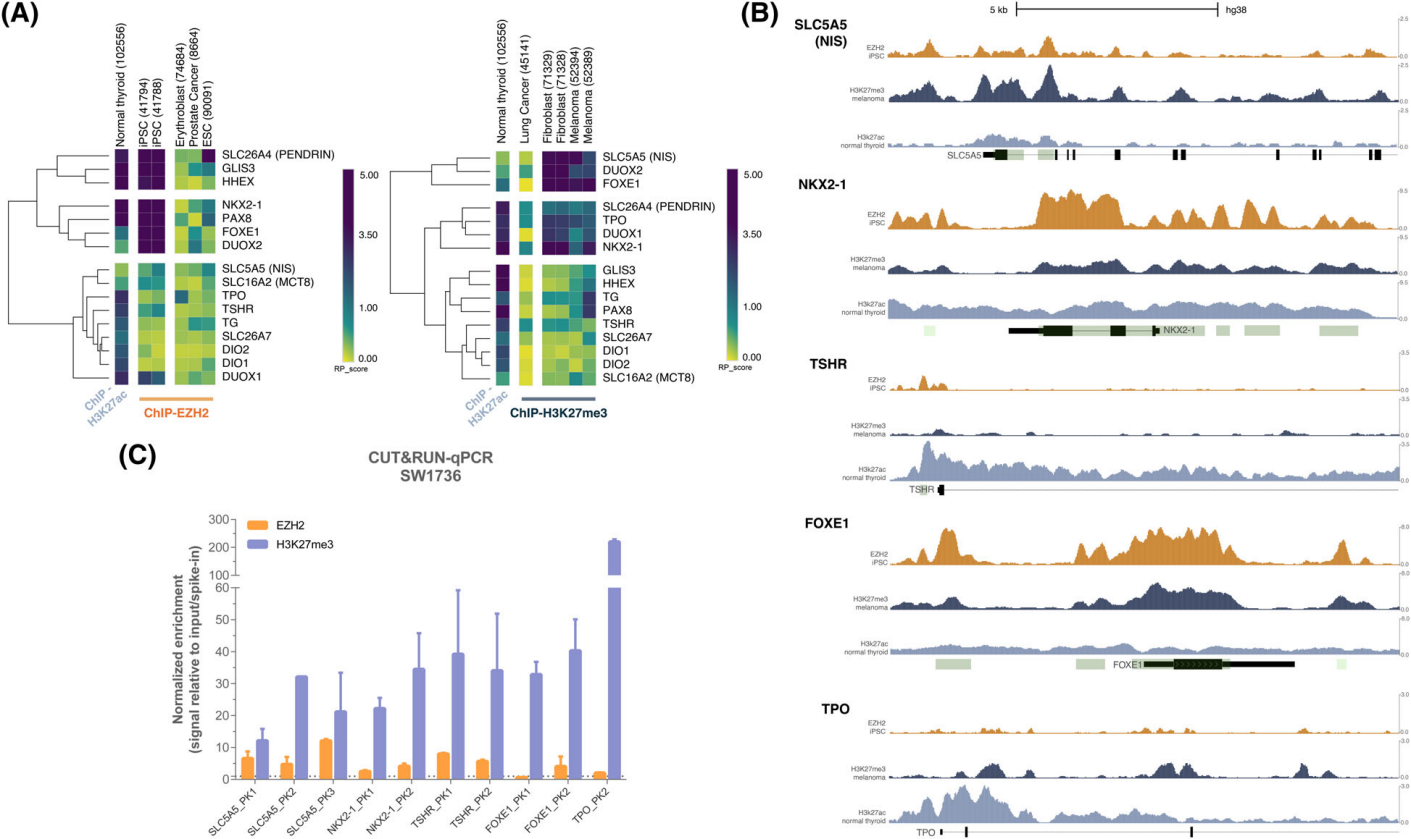
EZH2 regulates thyroid differentiation genes transcription through H3K27me3 deposition. (A) Heatmaps showing predicted EZH2 (left) and H3K27me3 (right) enrichment at candidate thyroid differentiation gene (TDG) loci obtained from public ChIP‐seq datasets available in the Cistrome Data Browser. Data were derived from non‐thyroid cell lines that do not express TDGs and integrated with H3K27ac regulatory potential scores (RP_score) from normal thyroid tissue to identify regions that are normally accessible and active in physiological conditions. Color scale represents regulatory potential, ranging from yellow (low) to dark blue (high). Hierarchical clustering using Euclidean distance was applied to group genes according to their enrichment profiles, highlighting clusters with the highest EZH2/H3K27me3 occupancy. (B) Genome browser tracks displaying representative ChIP‐seq peaks for EZH2 (orange, iPSC), H3K27me3 (dark blue, melanoma), and H3K27ac in normal thyroid tissue (light blue). Shown loci correspond to genes within highly enriched clusters that also respond to EZH2 inhibition: *SLC5A5*, *NKX2‐1*, *TSHR*, *FOXE1*, and *TPO*. (C) CUT&RUN‐qPCR validation of EZH2 and H3K27me3 enrichment at selected TDGs in SW1736 cells. Data are presented as mean ± SD for signal relative to input and normalized to spike‐in control.

To quantify the regulatory potential of EZH2/H3K27me3 at TDG loci, we used the highest enrichment scores from each dataset (Fig. 2A). Hierarchical clustering revealed strong EZH2 and/or H3K27me3 enrichment at multiple TDGs, particularly in iPSCs (EZH2) and fibroblast or melanoma cells (H3K27me3). Genes involved in iodine metabolism, including *SLC5A5* (NIS), *SLC26A4* (Pendrin), *DUOX2/1*, and *TPO*, as well as thyroid‐specific transcription factors *NKX2‐1*, *FOXE1*, *HHEX*, *PAX8*, and *GLIS3* showed a strong enrichment (Fig. 2A). Some genes, such as *NKX2‐1*, *FOXE1*, and *HHEX*, exhibited near‐complete occupancy by EZH2 and H3K27me3 (Fig. 2B, Fig. S5). Enriched regions frequently overlapped CpG islands, consistent with canonical PRC2 recruitment mechanisms.

To experimentally validate the predicted enrichment of EZH2 and H3K27me3 at TDGs, we performed CUT&RUN assays in the SW1736 cell line, which exhibits high EZH2 levels (Fig. S1). We immunoprecipitated EZH2 and H3K27me3 to recover enriched chromatin regions and quantified enrichment at different loci of *SLC5A5*, *NKX2‐1*, TSH receptor (*TSHR*), *FOXE1*, and *TPO* (Fig. 2B, Table 3)—genes that are transcriptionally activated following pharmacological inhibition of EZH2 methyltransferase (Fig. 1B, Fig. S3). Consistent with our hypothesis, we found a robust enrichment of both EZH2 and H3K27me3 marks at these loci in SW1736 cells (Fig. 2C, Fig. S6A), with H3K27me3 signals consistently higher than those of EZH2, reflecting a strongly repressed chromatin state. Additional ChIP‐qPCR assays confirmed EZH2/H3K27me3 enrichment at *SLC5A5* and *NKX2‐1* (Fig. S6B), further supporting a direct role for EZH2/PRC2‐mediated repression of thyroid differentiation genes in ATC.

## Discussion

In this study, we examined how PRC2‐driven epigenetic remodeling contributes to the silencing of thyroid differentiation genes in ATC cells, promoting cell dedifferentiation. Key genes like *SLC5A5* (NIS), *TPO*, and *TG* are essential for thyroid function—iodide uptake (SLC5A5/NIS) and hormone production (TPO, TG)—and are regulated by transcription factors PAX8, NKX2‐1, and FOXE1, all of which are frequently silenced in ATC. This loss of TDGs expression is a hallmark of ATC and contributes to its resistance to radioiodine therapy [21]. Previous studies have implicated EZH2/PRC2 activation in thyroid dedifferentiation, for example, by showing that EZH2 binds and represses the *PAX8* promoter [16], or that EZH2 inhibition restores TDGs expression and radioiodine uptake in PTC cells [17].

Our study extends these findings by identifying novel loci where EZH2 deposits the H3K27me3 repressive mark at the *SLC5A5*, *NKX2‐1*, *TSHR*, *FOXE1*, and *TPO* genes in ATC cells. Additionally, predictive analysis in non‐thyroid cell lines (that do not express thyroid‐related genes) revealed further enrichment of EZH2/H3K27me3 in other TDGs, including *SLC26A4* (Pendrin), *GLIS3*, *HHEX*, and *DUOX1/2*.

Functionally, we demonstrated that the treatment with a single agent, the US Food and Drug Administration (FDA)‐approved EZH2 inhibitor EPZ6438 (Tazemetostat), robustly restored TDG expression and significantly enhanced iodide uptake in ATC cell lines harboring either BRAF^V600E^ or RAS mutations. The observed 2.39‐fold to 6.22‐fold increases in uptake surpass those reported in previous studies using HDAC (histone deacetylase) inhibitors in combination with VCP or AP2 inhibitors in undifferentiated thyroid cancer models [22, 23].

Interestingly, while prior work in PTC cells showed that dual inhibition of MAPK and EZH2 improves iodide uptake [17], our study shows that this strategy is also effective in ATC. Both BRAF^V600E^ and RAS‐mutated ATC cells (NRAS^Q61R^ and HRAS^G13R^) responded to EZH2 inhibition alone, and further upregulation of TDGs was observed when combined with MEK1/2 inhibition. Although the direct impact on iodide uptake remains to be evaluated, these results suggest a functional crosstalk between EZH2 and the MAPK pathway, as EZH2 expression was partly dependent on the MAPK pathway. Indeed, this is consistent with a previous study from breast cancer cells where EZH2 levels decreased following MAPK inhibition [24].

Regarding the therapeutic context of ATC, the FDA recently approved the combination of dabrafenib (RAF inhibitor) plus trametinib (MEK inhibitor) as a neoadjuvant strategy for treating BRAF^V600E^‐mutated ATC and other unresectable or metastatic BRAF^V600E^‐mutant solid tumors [25]. Furthermore, combining pembrolizumab (anti‐PD‐L1) with dabrafenib and trametinib prior to surgery has shown promising results, with modest improvements in median overall survival in clinical trials [26, 27]. Of interest, combined RAF plus MEK provides greater clinical benefit and higher response rates than RAF inhibition alone [25]. In cases that harbor BRAF mutations (40–45% of ATCs). In contrast, RAS mutations (10–20% of ATCs) are associated with poorer outcomes [11, 12, 28, 29], largely due to the absence of effective targeted therapies in thyroid cancer, highlighting the therapeutic relevance of our findings.

Finally, this study proposes a mechanism for thyroid cell dedifferentiation mediated by EZH2/PRC2 through deposition of H3K27me3. Previous studies have demonstrated that chromatin remodeling, histone modifications, and DNA methylation all influence *SLC5A5* transcription and function. For instance, HDAC inhibition has repeatedly been shown to enhance NIS levels and radioiodine uptake [30, 31], with Vorinostat (SAHA, FDA‐approved) being particularly effective in both well‐differentiated and undifferentiated thyroid cancer models when combined with chloroquine or VCP inhibitors to prevent NIS internalization and degradation [22, 23]. In addition to histone modifications, DNA regulatory regions of *SLC5A5* were identified as methylation targets [32]. In fact, hypermethylation of *SLC5A5* distal enhancer has been reported in thyroid tumors [33] and treatment with the demethylating agent 5‐aza‐2′‐deoxycytidine improved NIS expression and radioiodine uptake in FTC cells [33], though ineffective in ATC cells [34]. In this context, the EZH2‐mediated repression of multiple TDGs may represent an additional and targetable layer of control contributing to thyroid dedifferentiation and loss of iodide uptake.

In conclusion, our findings highlight a pivotal role of EZH2/PRC2 in the epigenetic repression of thyroid differentiation genes. First, we show that EZH2 directly binds to TDG and deposits the repressive H3K27me3 mark, silencing their transcription. Second, the pharmacological inhibition of EZH2 activity with the FDA‐approved drug Tazemetostat (EPZ6438) restores iodine trapping, a therapeutic hallmark lost in ATC. Third, combining EPZ6438 with MEK1/2 inhibition further enhances this effect, suggesting a promising repurposing and neoadjuvant therapeutic strategy for ATC, including the particularly challenging RAS‐mutated cases.

## Author contributions

DCM, conceptualization and design of the study, methodology, data curation, formal analysis, writing (original draft and editing). MMC, ChIP‐qPCR assay methodology and analysis. GH, VGR, and CS‐N, iodide uptake assay methodology and curation. ETK, resources, funding, supervision. CSF, conceptualization, design, resources, funding, methodology, data curation, writing (original draft and editing), review, supervision, project administration.

## Supporting information


**Fig. S1.** EZH2 is overexpressed in BRAF‐mutated and RAS‐mutated anaplastic thyroid cancer cells.
**Fig. S2.** EZH2 and MEK1/2 targeting improves thyroid differentiation genes in RAS‐mutated anaplastic thyroid cancer cells.
**Fig. S3.** EZH2 and MEK1/2 targeting improves thyroid differentiation genes in BRAF‐mutated anaplastic thyroid cancer cells.
**Fig. S4.** Non‐radioactive iodide uptake in nontumoral and thyroid cancer cells.
**Fig. S5.** EZH2 and the repressive H3K27me3 mark are enriched at additional thyroid differentiation genes.
**Fig. S6.** EZH2 deposits H3K27me3 mark in order to repress thyroid differentiation genes in anaplastic thyroid cancer.

## Data Availability

This study used publicly available ChIP‐seq data derived from the Cistrome data browser at http://cistrome.org/db/ (version 2.0; 12/12/2024). The respective resources are listed in the methods section. The additional data that support the findings of this study are available within the article and the Supporting Information.
