# Supplementary material for: Targeting EZH2 reverses thyroid cell dedifferentiation and enhances iodide uptake in anaplastic thyroid cancer
Source: FEBS Lett. 2025 Oct 28;600(2):215–25. doi: 10.1002/1873-3468.70207 (PMC12834004; doi:10.1002/1873-3468.70207)
Supplement: Supplementary file 1 — Fig. S1. EZH2 is overexpressed in BRAF‐mutated and RAS‐mutated anaplastic thyroid cancer cells. Fig. S2. EZH2 and MEK1/2 targeting improves thyroid differentiation genes in RAS‐mutated anaplastic thyroid cancer cells. Fig. S3. EZH2 and MEK1/2 targeting improves thyroid differentiation genes in BRAF‐mutated anaplastic thyroid cancer cells. Fig. S4. Non‐radioactive iodide uptake in nontumoral and thyroid cancer cells. Fig. S5. EZH2 and the repressive H3K27me3 mark are enriched at additional thyroid differentiation genes. Fig. S6. EZH2 deposits H3K27me3 mark in order to repress thyroid differentiation genes in anaplastic thyroid cancer. [file FEB2-600-215-s001.docx]

**SUPPLEMENTARY INFORMATION**

**Targeting EZH2 reverses thyroid cell dedifferentiation and enhances iodide uptake in anaplastic thyroid cancer**

Diego Claro de Mello ^(1)^, Marcella Maringolo Cristovão ^(1)^, Guilherme Henrique ^(2, 3)^, Vinicius Gonçalves Rodrigues ^(2, 3)^, Caroline Serrano-Nascimento ^(2, 3)^, Edna Teruko Kimura ^(1)^, Cesar Seigi Fuziwara ^(1)^*

^(1)^ Department of Cell and Developmental Biology, Institute of Biomedical Sciences, University of São Paulo (USP), CEP 05508-000, São Paulo/SP, Brazil

^(2)^ Department of Medicine, Laboratory of Molecular and Translational Endocrinology Medicine, Federal University of São Paulo (UNIFESP), CEP 04039-032, São Paulo/SP, Brazil

^(3)^ Institute of Environmental, Chemical and Pharmaceutical Sciences (ICAQF), Department of Biological Sciences, Federal University of São Paulo (UNIFESP), CEP 09972-270, Diadema/SP, Brazil

* Author to whom correspondence should be addressed.

**Running title:** **EZH2 inhibition enhances iodide uptake in ATC**

**Keywords**: anaplastic thyroid cancer, EZH2, thyroid differentiation, Tazemetostat, iodide uptake, NIS, RAS mutation, BRAF mutation

**
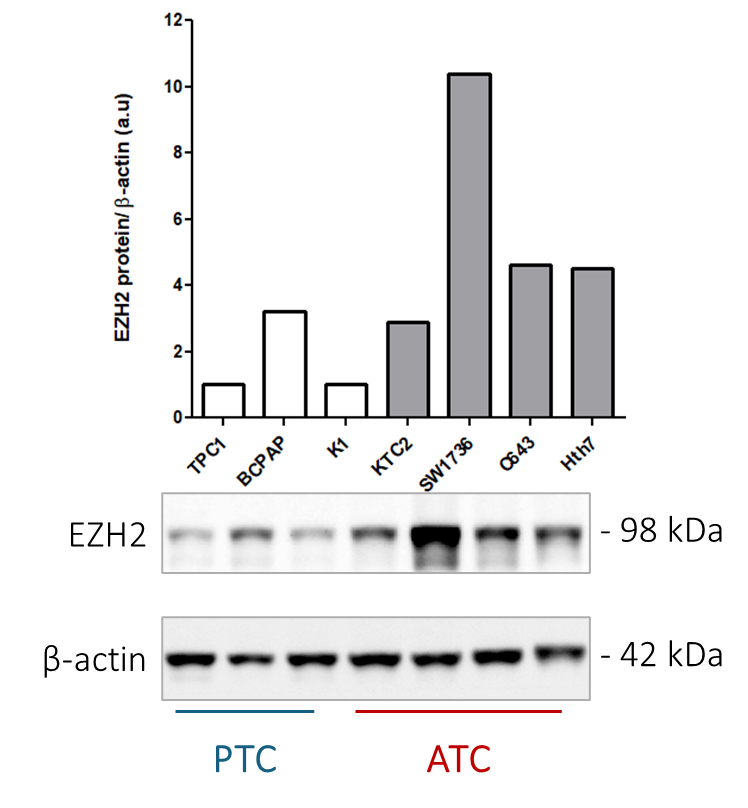
**

**Figure S1**. **EZH2 is overexpressed in BRAF-mutated and RAS-mutated** **anaplastic thyroid cancer cells.** Western blot of EZH2 and β-actin (loading control) in PTC cells (TPC1, BCPAP, K1) and ATC cell lines with BRAF mutation (KTC2 and SW1736), and RAS mutations (C643 and Hth7). The graph is represented as normalized values against TPC1 cell lines (a.u, arbitrary units). The quantification was performed using the ImageJ software.

**
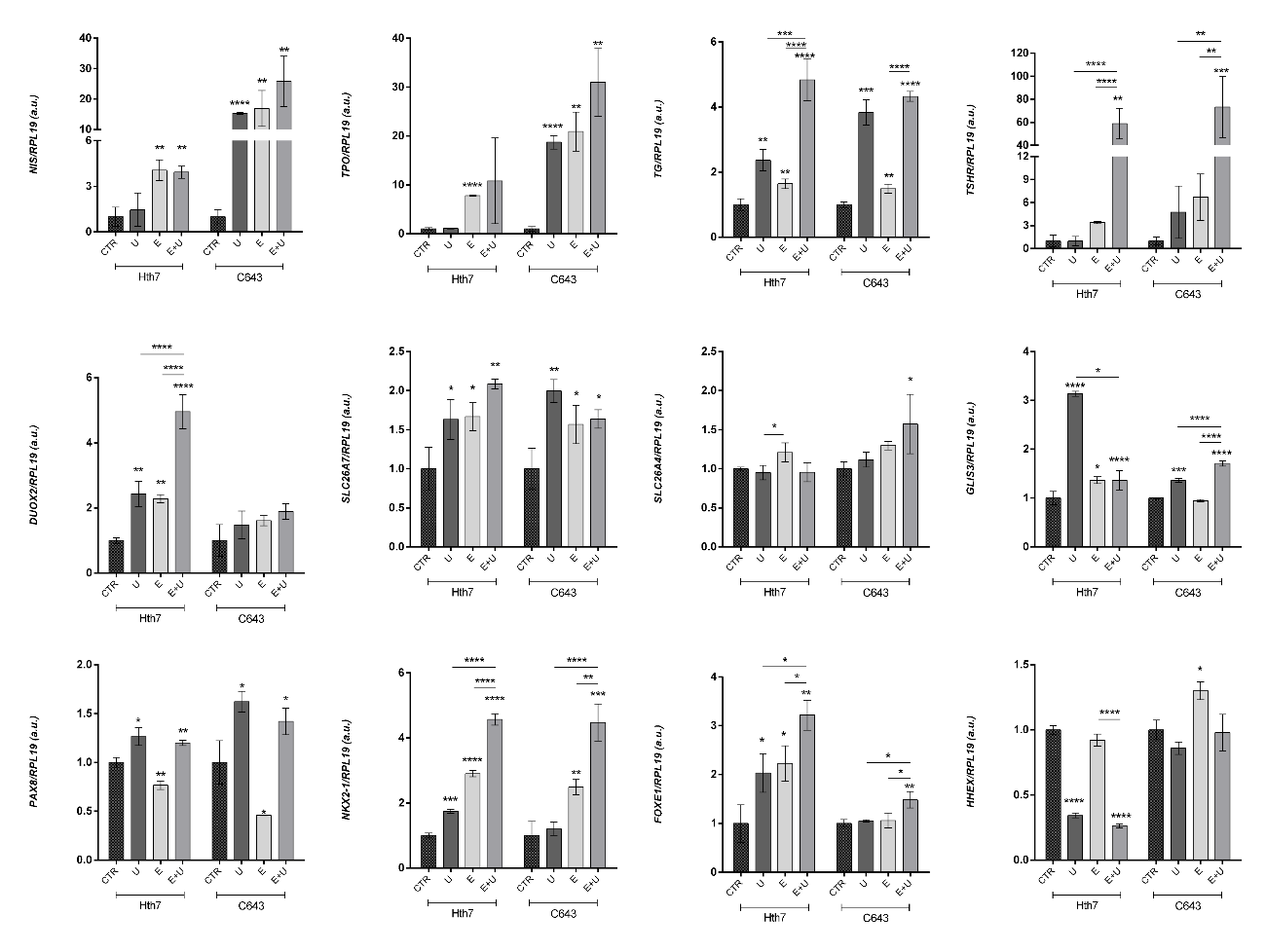
**

**Figure S2**. **EZH2 and MEK1/2 targeting improves thyroid differentiation genes in RAS-mutated anaplastic thyroid cancer cells.** Bar plot with statistical analysis of gene expression of thyroid-differentiation genes panel demonstrated in Figure 1B in Hth7 and C643 cells following treatment with U0126 (U, MEK1/2 inhibitor), EPZ6438 (EPZ, EZH2 inhibitor) or combined (E+U). Data is shown as mean ± SD (n = 3) for gene expression. a.u., arbitrary units; Statistical difference are ploted according to analysis of variance (ANOVA) and significative p values are shown as *, p < 0.05; **, p < 0.01; ***, p < 0.001; ****, p < 0.0001 vs. DMSO-control (CTR).

**
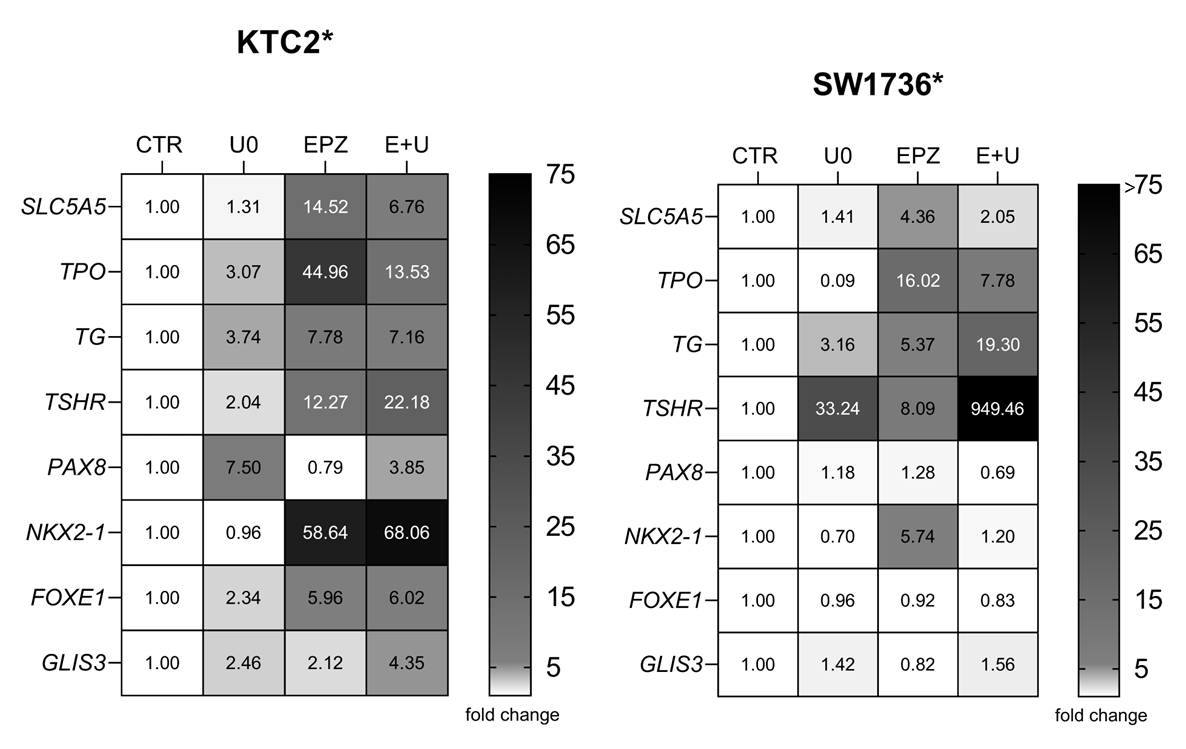
**

**Figure S3**. **EZH2 and MEK1/2 targeting improves thyroid differentiation genes in BRAF-mutated anaplastic thyroid cancer cells.** Heatmap demonstration of previously published results of qPCR of gene expression of 8 thyroid differentiation genes (*SLC5A5*, *TPO*, *TG*, *TSHR*, *PAX8*, *NKX2-1*, *FOXE1* and *GLIS3*) in KTC2 and SW1736 cells treated with U0126 (U, MEK1/2 inhibitor), EPZ6438 (EPZ, EZH2 inhibitor) or combined (E+U). Values are expressed as fold change *vs.* DMSO-treated controls (CTR).

**
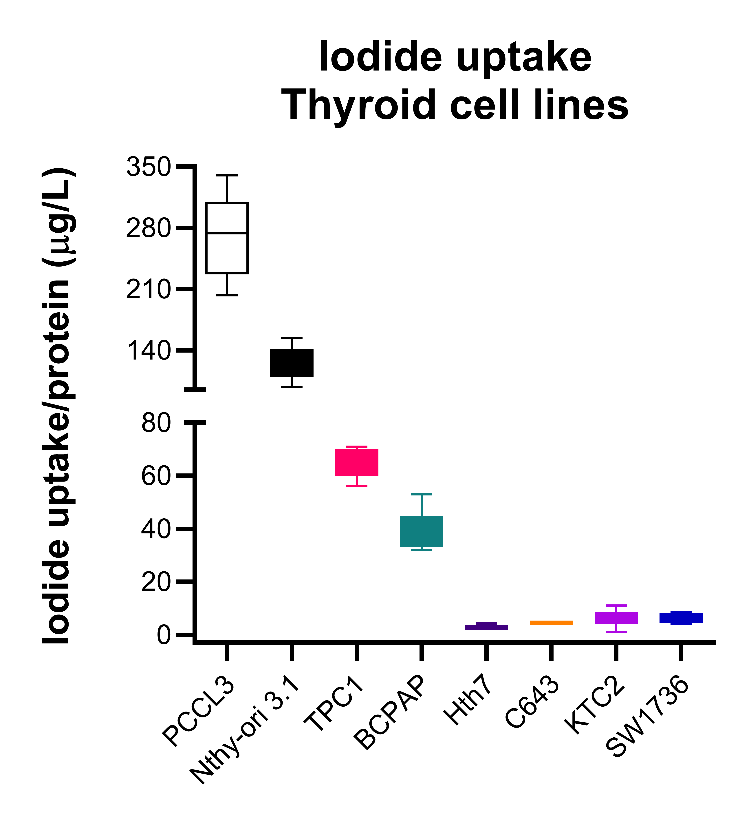
**

**Figure S4**. **Non-radioactive iodide uptake in nontumoral and thyroid cancer cells.** To validate the methodology for measuring NIS-mediated iodide uptake, we performed a non-radioactive uptake assay comparing nontumoral thyroid cell lines (PCCL3, normal rat thyrocytes; and Nthy-ori 3-1, human thyroid cells), PTC cell lines (TPC1 and BCPAP), RAS-mutated ATC cell lines (Hth7 and C643), and BRAF-mutated ATC cell lines (KTC2 and SW1736). The results show a progressive loss of NIS-mediated iodide trapping consistent with the loss of differentiation across tumor subtypes. Box plots represent mean ± SD from two independent experiments.

**
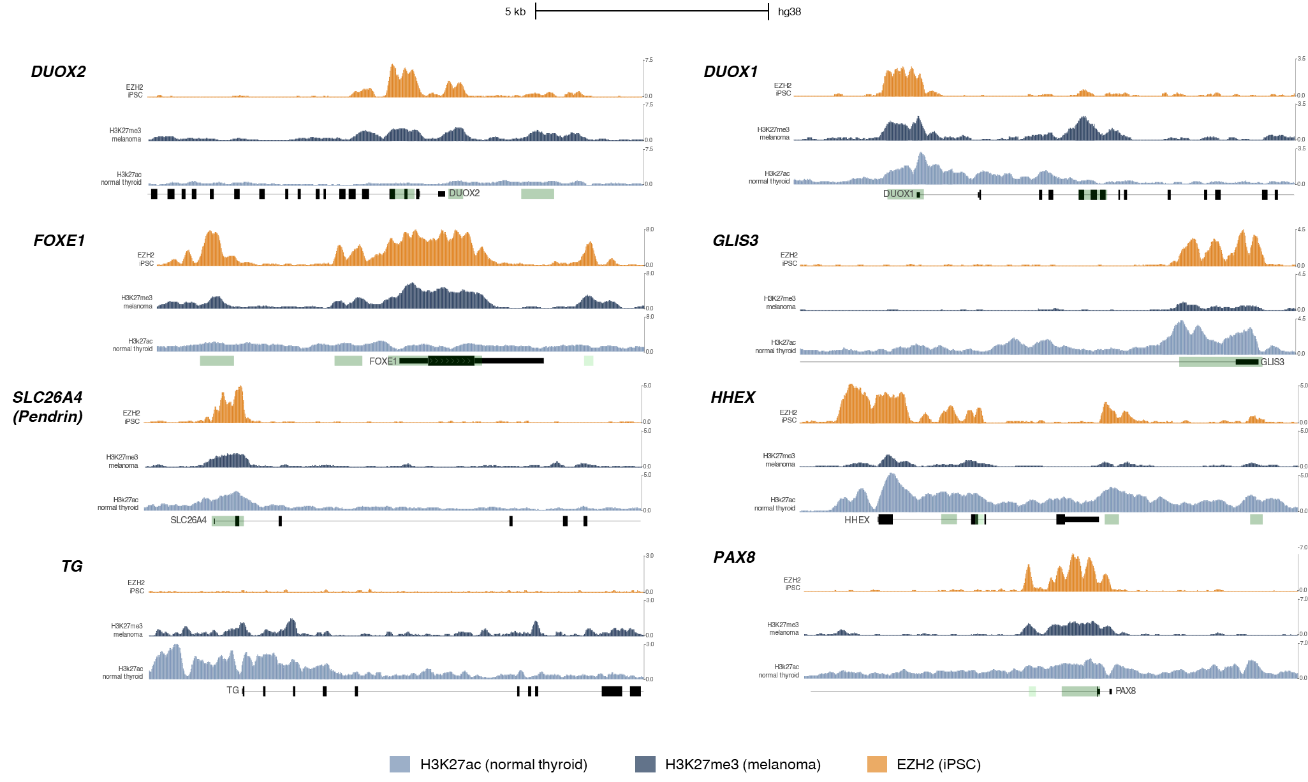
**

**Figure S5**. **EZH2 and the repressive H3K27me3 mark are enriched at additional thyroid differentiation genes.** Genome browser tracks displaying representative ChIP-seq peaks for EZH2 (orange, iPSC), H3K27me3 (dark blue, melanoma), and H3K27ac in normal thyroid tissue (light blue). Shown loci correspond to genes within enriched clusters: *DUOX2*, *SLC16A2*, *SLC26A4*, *TG*, *DUOX1*, *GLIS3*, *HHEX* and *PAX8*.

**
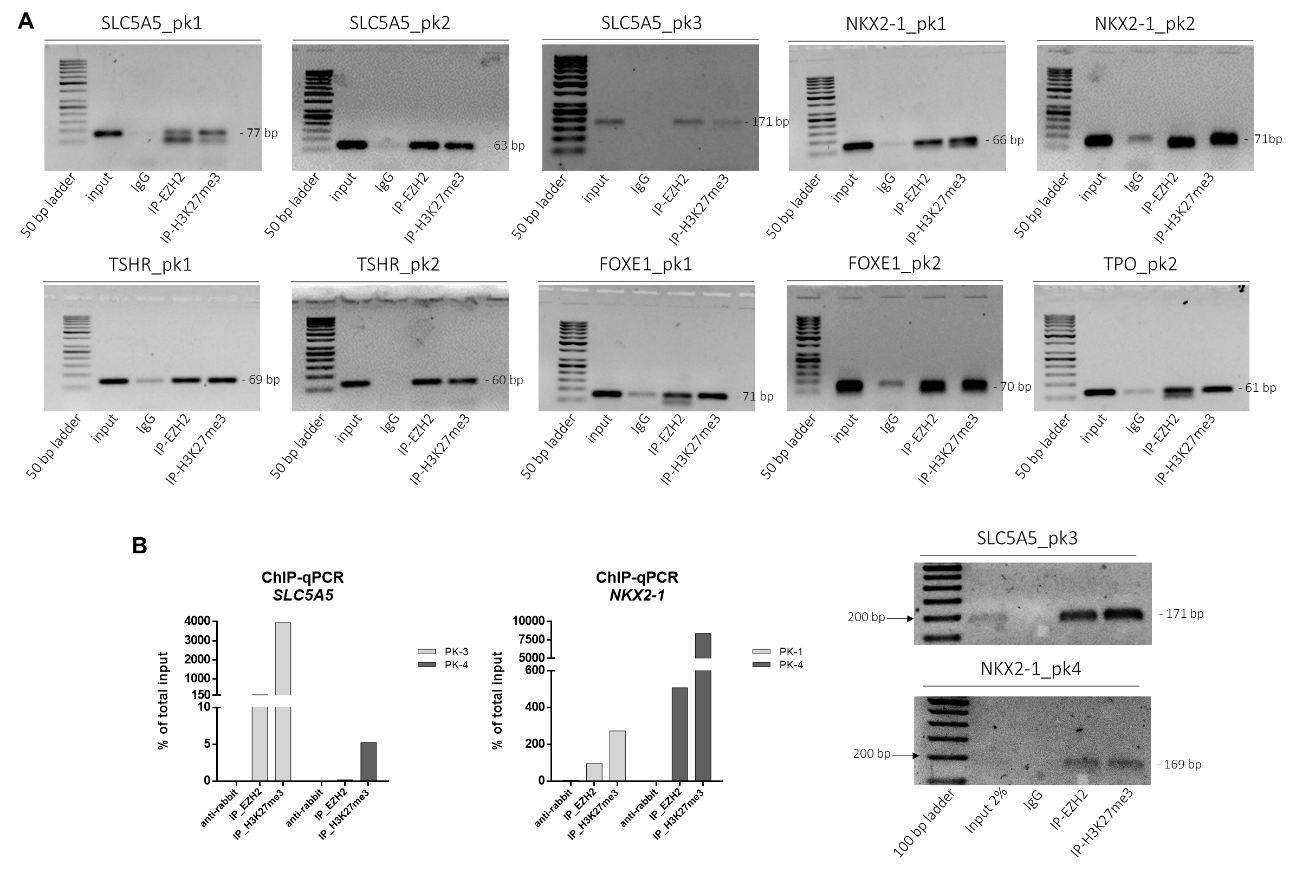
**

**Figure S6**. **EZH2 deposits H3K27me3 mark in order to repress thyroid differentiation genes in anaplastic thyroid cancer. A.** Representative 2% agarose gels showing CUT&RUN-qPCR products from SW1736 cells using antibodies against EZH2 or H3K27me3, compared with non-enriched DNA input and an IgG negative control. The first lane contains the 50 bp DNA ladder, and the expected amplicon sizes and corresponding peaks are indicated. **B.** ChIP-qPCR validation of EZH2 and H3K27me3 enrichment at selected peaks within SLC5A5 and NKX2-1 loci in SW1736 cells, with representative agarose gels shown below. bp, base pairs; pk, peak.
